# Targeting of G-protein coupled receptor 40 alleviates airway hyperresponsiveness through RhoA/ROCK1 signaling pathway in obese asthmatic mice

**DOI:** 10.1186/s12931-023-02361-1

**Published:** 2023-02-17

**Authors:** Xixi Lin, Like Wang, Xiaojie Lu, Yuanyuan Zhang, Rongying Zheng, Ruijie Chen, Weixi Zhang

**Affiliations:** 1 Department of Pharmacy, The Second Affiliated Hospital and Yuying Children’s Hospital of Wenzhou Medical University, Wenzhou, 325027 Zhejiang China; 2 Department of Pediatric Allergy and Immunology, The Second Affiliated Hospital and Yuying Children’s Hospital of Wenzhou Medical University, Wenzhou, 325027 Zhejiang China; 3 School of Pharmaceutical Sciences, Wenzhou Medical University, Wenzhou, 325027 Zhejiang China

**Keywords:** GPR40, Asthma, Obesity, RhoA/ROCK1, Airway hyperresponsiveness

## Abstract

Obesity increases the severity of airway hyperresponsiveness (AHR) in individuals with asthma, but the mechanism is not well elucidated. G-protein coupled receptor 40 (GPR40) has been found to induce airway smooth muscle contraction after activated by long-chain fatty acids (LC-FFAs), suggesting a close correlation between GPR40 and AHR in obese. In this study, C57BL/6 mice were fed a high-fat diet (HFD) to induce obesity with or without ovalbumin (OVA) sensitization, the regulatory effects of GPR40 on AHR, inflammatory cells infiltration, and the expression of Th1/Th2 cytokines were evaluated by using a small-molecule antagonist of GPR40, DC260126. We found that the free fatty acids (FFAs) level and GPR40 expression were greatly elevated in the pulmonary tissues of obese asthmatic mice. DC260126 greatly reduced methacholine-induced AHR, ameliorated pulmonary pathological changes and decreased inflammatory cell infiltration in the airways in obese asthma. In addition, DC260126 could down-regulate the levels of Th2 cytokines (IL-4, IL-5, and IL-13) and pro-inflammatory cytokines (IL-1β, TNF-α), but elevated Th1 cytokine (IFN-γ) expression. In vitro*,* DC260126 could remarkedly reduce oleic acid (OA)-induced cell proliferation and migration in HASM cells. Mechanistically, the effects that DC260126 alleviated obese asthma was correlated with the down-regulation of GTP-RhoA and Rho-associated coiled-coil-forming protein kinase 1 (ROCK1). Herein, we proved that targeting of GPR40 with its antagonist helped to mitigate multiple parameters of obese asthma effectively.

**Supplementary Information:**

The online version contains supplementary material available at 10.1186/s12931-023-02361-1.

## Introduction

Asthma is a serious chronic and heterogeneous airway inflammatory disease, whose core characteristics include airway hyperresponsiveness (AHR), reversible airway obstruction, and remodeling of the airways [1]. Obesity, the end result of a mismatch between energy intake and energy expenditure, is rising at an alarming rate in the worldwide, which poses a substantial threat to public health and takes a great toll on health care costs [2, 3]. Obesity has been well recognized as an important comorbidity in patients with asthma, representing an unique phenotype and endotype [4]. A published study involving over 300,000 subjects found a close link between asthma and obesity, suggesting the risk of asthma increased with increasing body weight [5]. Although, the association of asthma and obesity has been demonstrated in numerous epidemiological studies, the exact underlying mechanisms and detailed pathogenic link are not well understood. Currently, using corticosteroids to inhibit airway inflammation, or in combination with bronchodilators and anticholinergic drugs to relieve constricted airways are the mainstay in asthma therapy [6, 7], but asthma in the obese usually accompanied with a more severe disease outcome that does not respond as well to conventional therapy, underscoring the worrying problem of treating this population with therapies that were developed.

AHR is a hallmark feature of asthma and provides a quantitative measure of disease severity [8]. It has been shown that obesity can cause substantial changes to the mechanics of the lungs, and lead to asthma-like symptoms such as wheeze, and AHR [9]. In mice, obesity has been shown to induce AHR, for instance, to up-regulate allergen-induced AHR or enhance ozone-induced AHR [10, 11]. Nevertheless, obese asthma is associated with more severe AHR [12]. It is generally accepted that airway smooth muscle (ASM) is the key end effector of acute airway narrowing, and figure out the contraction of the ASM is a critical event behind an observation of AHR [13]. Recent evidences have suggested that the increased ASM mass is associated with severe asthma phenotype, and, on the other hand, is correlated with a decreased lung function [14]. Therefore, targeting ASM in obese asthma and developing new pharmacologic strategies for limiting ASM mass and contraction maybe of great therapeutic value.

G-Protein Coupled Receptors (GPCRs) are seven-transmembrane (7 TM) receptors that constitute the largest family of drug targets in humans [15]. Activation or inactivation GPCRs comprise mainstay asthma therapies, thus, identifying a novel GPCR agonist or antagonist become a promising approach to develop newer and effective anti-asthma drugs [16]. GPR40, is an orphan G-protein coupled receptor that has medium- and long-chain fatty acids as ligands [17]. GPR40 has been identified on the human airway and proved to be able to induce cell proliferation [18]. Imporantly, previous studies have also identified the expression of GPR40 in the airway smooth muscle (ASM) [19]. The free fatty acids (FFAs) level was increased in most obese patients, activation of GPR40 is capable of inducing HASM cells proliferation and promoting smooth muscle contraction, suggesting a pivotal role in obesity-associated AHR [20].

Homolog family member A (RhoA) is a member of the Rho family small GTPases, which could switch by alternating between GDP-RhoA (inactive state) and GTP-RhoA (active state) [21]. Active GTP-bound RhoA activates ROCK, participating in the regulation of smooth muscle contractions through the mechanism called calcium sensitization [22]. RhoA/ROCK1 signal pathway has been reported to serve as a proximal downstream effector of numerous GPCRs, and plays an important role in the pathophysiology of asthma, including airway smooth muscle contraction, AHR, and airway remodeling [23]. Importantly, a great number of studies have demonstrated that ROCK1 isoform plays an inhibitory role for the regulation of diseases in diet-induced obesity, including insulin resistance [24], fatty liver diseases [25], and atherosclerosis [26]. However, whether GRP40 regulates AHR in obese asthma through RhoA/ROCK1 signal pathway remains elusive.

Herein, we tested the hypothesis that blockage of GRP40 expression with DC260126 may help relieve AHR in obese asthma and inhibited cell proliferation and migration in HASM cells in vitro. Mechanistically, we attempted to identify the signaling pathways that are involved in the inactivation of RhoA and its downstream, ROCK1. Our research offers a novel perspective on obese asthma pathogenesis, and it is possible to provide a new therapeutic intervention for this respiratory disease.

## Materials and methods

### Reagents

DC260126 (MCE, New Jersey, USA) was employed to antagonize GPR40 expression. Y-27632, the inhibitor of ROCK, was obtained from Tocris Bioscience (Bristol, UK). High-fat diet (NO. MD12032) was purchased from Medicience Ltd (Jiangsu, China). Methacholine (Mch), Ovalbumin (OVA) and DMSO were bought from Sigma-Aldrich (St. Louis, MO), TRIzol reagents were used to extract total RNA (Takara, Otsu, Shiga, Japan). Oleic acid (OA) was purchased from Sigma-Aldrich Company, USA. The following antibodies: RhoA (1:5000, #AB187027, rabbit polyclonal, Abcam), ROCK1 (1:2000, #ab45171, rabbit polyclonal, Abcam), GPR40 (1:1000, #DF2745, rabbit polyclonal, Affinity Biosciences), GAPDH (1:5000, #AF7021, rabbit polyclonal, Affinity Biosciences) were applied to the western blot analysis. Mouse FFAs, IL-4, IL-5, IL-13, IL-1β, TNF-α, and IFN-γ ELISA kits were obtained from eBioscience (San Diego, CA).

### Cell culture

Human airway smooth muscle (HASM) cells were sourced from the ScienCell Research Laboratories (Cat. No. 3410, Carlsbad, CA, USA). According to the manufacturer’s instructions, the cell type was confirmed by immunofluorescence with antibodies specific to α-smooth muscle actin. Cells between passages 3 and 10 were used for the subsequent experiments. HASM cells were grown in Smooth Muscle Cells Medium (SMCM) supplemented with 2% FBS, 1% smooth muscle cell growth supplement (5 ng/ml rh FGF, 5 µg/ml insulin, 50 µg/ml ascorbic acid, 5 µg/ml Transferrin, 10 mM L-glutamine, and 5 ng/ml rh EGF ng/ml), 10 mM HEPES buffer and 1% penicillin/ streptomycin, and were incubated at 37 °C in a humidified atmosphere containing 5% CO2.

### Animals

Male C57BL/6 mice (3–4 weeks old, weighing 20 ± 2 g), were purchased from Zhejiang Experimental Animal Center (No. SCXK 2019-0002) and housed in Plexiglas cages under 12/12 h light/dark cycle, and given food and water ad libitum in the Laboratory Animal Center of Wenzhou Medical University. The Committee of the Ethics of Animal Experiments of the Wenzhou Medical University (license number: wydw2019-0223) approved this study. All the animals in our study received humane care.

Animals were randomly selected and allocated to two groups: the lean group, and the obesity group. The obesity model was established by feeding mice with high fat diet (HFD) (MD12032, Medicience Ltd, China) that containing 45% kcal from fat for 16 weeks, the lean mice were fed a normal chow diet (MD12031, Medicine Ltd, China) that containing 10% kcal from fat as the control. In order to establish an asthma model in lean or obese mice, mice were sensitized by i.p. injection of 10 μg OVA (Sigma, United States) emulsified in 20 mg Al (OH)_3 _gel in 0.1 mL normal saline on day 1 and 13 starting from 12th week, and then challenged with 10 mg/ml aerosolized OVA for 30 min a day for 7 consecutive days. The control mice were sensitized and challenged with 0.9% NS.

### Treatment with DC260126 or Y-27632 in obese asthmatic mice

**Fig. 1:**
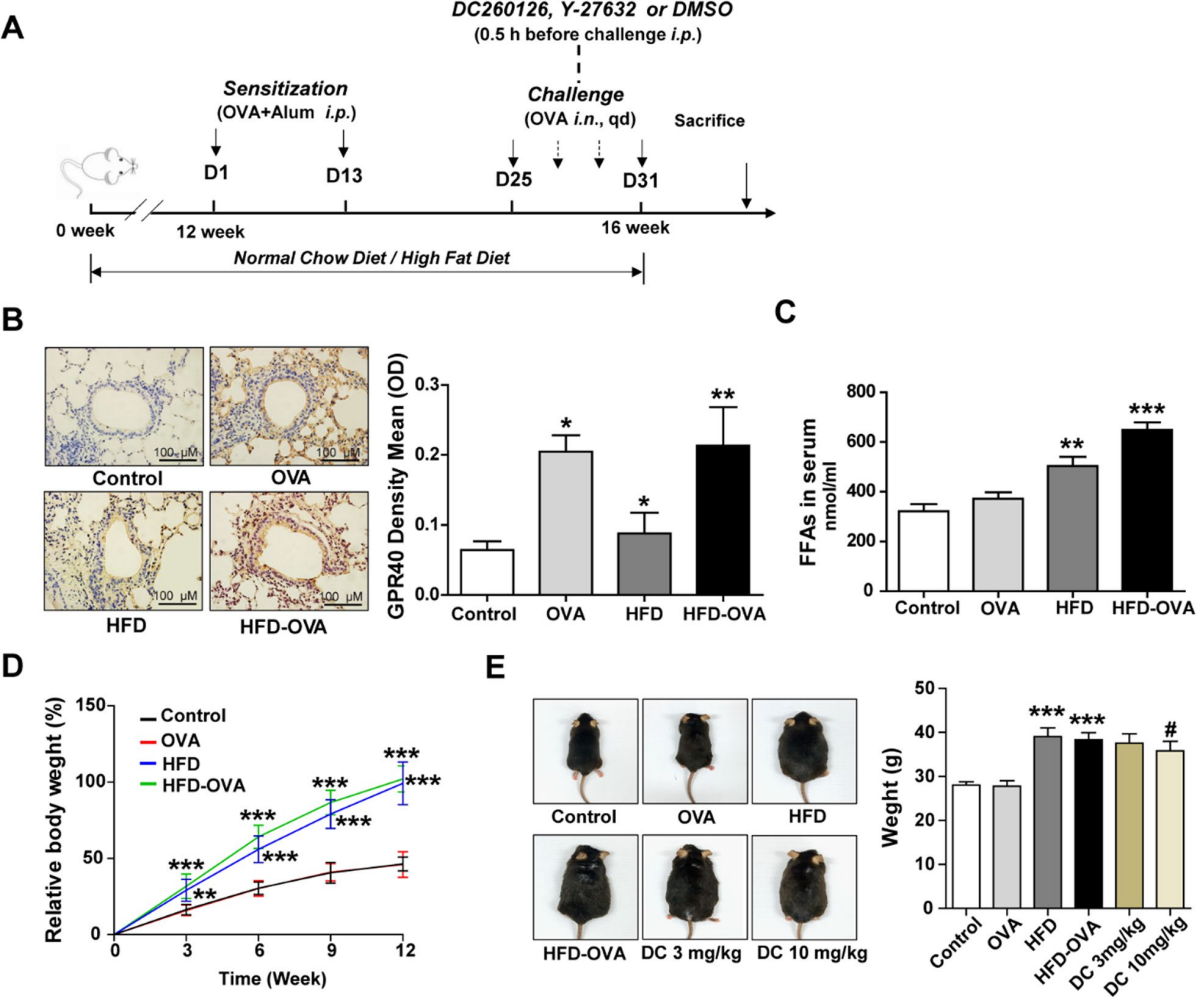
GPR40 expression is elevated in the lung tissues of obese asthmatic mice. **A** The mice were fed with normal chow diet or HFD for 12 weeks, followed by sensitized with OVA that emulsified in Al (OH)_3_ gel on day 1 and 13 from the 12th week, and then challenged with aerosolized OVA for 0.5 h a day from day 25 to 31. DC260126 was given to HFD-OVA model mice daily by i.p. injection at 0.5 h before each challenge, vehicle (0.1% DMSO) was given to the mice in control, OVA, HFD, and HFD-OVA instead. **B** The GPR40 expression in the pulmonary tissues of mice was assessed by immunohistochemical staining. The data were represented as the mean ± S.E.M. *P < 0.05, and **P < 0.01 compared with the control group. **C** FFAs level in serum was assessed by ELISA. **D** The body weight in lean mice (Control, OVA group) and obese mice (HFD, HFD-OVA group) was recorded every three weeks, the results were expressed as the percentage of body weight gain. **E** Mice body weight was measured with (HFD + OVA + DC260126 3 mg/kg, HFD + OVA + DC260126 10 mg/kg group) or without (Control, OVA, HFD, HFD-OVA group) DC260126 administration during 7 consecutive days. The data were represented as the mean ± S.E.M. (n = 6 per group). **P < 0.01 and ***P < 0.001 compared with the control group. ^#^*P* < 0.05 compared with the HFD-OVA group

The lean and HFD-induced obese mice were randomly divided into 6 groups (n = 6): the control group (Normal chow diet), the OVA group (Normal chow diet + OVA), the HFD group (High fat diet), the HFD-OVA group (High fat diet + OVA), the DC260126 3 mg/kg group (High fat diet + OVA + DC260126 3 mg/kg) and DC260126 10 mg/kg group (High fat diet + OVA + DC260126 10 mg/kg). Herein, we selected DC260126 at 3 and 10 mg/kg as the intervention dose was based on our pretest that presented in Additional file 1: Fig. S1, for we found DC260126 at high dose (30 mg/kg) failed to exert a stronger lowering effect as expected, but probably aggravated the neutrophilic airway inflammation in asthma. DC260126 (DC), a GPR40 antagonist, was dissolved in DMSO (20 mg/ml) and stored at − 20 °C. Schedule of study design was showed in Fig. 1A, briefly, DC260126 was administered intraperitoneally for 7 consecutive days, 30 min before every OVA challenge in obese mice. 0.1% DMSO treatments were performed to the mice in the control and OVA model group to eliminate the interference errors that caused by the vehicle. Y-27632, a ROCK antagonist, was dissolved in 0.9%NS and stored at − 20 °C. Y-27632 at 3 mg/kg or 10 mg/kg was administered intraperitoneally to model mice refer to the schedule as described above.

### Determination of airway hyperresponsiveness (AHR)

Airway hyperresponsiveness (AHR) was determined following our previous research [12] by measuring changes in the airway resistance (R_rs,_ cmH_2_O/ml/s) after challenged with aerosolized methacholine (Mch) in a concentration gradient (3.125, 6.25, 12.5, 25 and 50 mg/mL) using the FlexiVent system (SCIREQ, Quebec, Canada). Anesthetized (1% pentobarbital sodium 50 mg/kg i.p.), tracheostomized (stainless-steel cannula, 12 G) mice were mechanically ventilated (at 150 breaths/min, with a tidal volume of 10 ml/kg, positive end-expiratory pressure of 3 cmH_2_O). Rrs was measured at every dose to calculate the percentage of each concentration to baseline. The baseline values of Rrs were measured at the stage of aerosolized saline nebulizing.

### Bronchoalveolar lavage fluids (BALFs) preparation and cell counts

After the left upper lobe lung was ligated, BALFs were obtained by flushing the right lung three times with 0.5 ml of phosphate buffer solution (PBS) containing 1% BSA and 5000 IU/L heparin. The sample was centrifuged and its supernatant was analyzed by ELISA. The pelleted BALF cells were resuspended in PBS, and the total number of leucocytes was counted using a Neubauer chamber according to our previous study [27]. An aliquot of 200 BALF cells were stained with Wright-Giemsa and differentially identified under a light microscope according to classical cell morphology. The total number of each cell type was determined by multiplying the percentage by the total number of cells. The results were expressed as the numbers of each type of cell population in BALFs (1 ml).

### Histopathological examination

Each mouse’s left middle lobe lung was collected and fixed in 4% paraformaldehyde. It was then embedded with paraffin, sliced into 3–4 μm sections, and examined with hematoxylin and eosin (H&E) staining. The severity of lung inflammation was determined under light microscopy and scored based on the following histologic grading system (scored 0–4) according to our previous study [12]. Collagen deposition around the bronchial airway was observed by Masson's trichrome staining. The severity of collagen deposition was evaluated using the Image Pro 6.1 software system, the results were expressed as collagen volume fraction (%) = area of collagen fiber/area of total view. Goblet cell hyperplasia was observed with PAS staining. The percentage of PAS staining-positive cells in the airway epithelium was quantified [28]. To perform the immunohistochemistry analysis, the experimental protocol in Streptavidin–Biotin Complex kit that bought from Boster Bio-engineering Ltd. Co., (Wuhan, China) was employed. The diluted primary anti-GPR40 (1:100) were incubated the slices overnight at 4 °C. Afterwards, the immunolabeling were visualized using 3,3′-diaminobenzidine (DAB) and analyzed by DP2-BSW software (Olympus, Tokyo, Japan). Semiquantitative evaluations of histopathological images in the present study were performed by a blinded observer.

### Cell proliferation assay

Cell proliferation assay was determined by Cell Counting Kit-8 (CCK-8, Beyotime Institute of Biotechnology, Shanghai, China) assay. A total of 100 µL of cell suspension (5000 cells/well) was pre-incubated for 24 h (at 37 °C, 5% CO2). After adding 10 µL of various concentrations of oleic acid with or without DC260126 pretreatment for 30 min, we incubated each well of the plates with 10 µL of CCK-8 solution for 3 h. The absorbance was measured at 450 nm by a microplate reader (Bio-Tek, Winooski, VT, USA).

### Real-time PCR

TRIzol reagent bought from Takara company was applied to extract total RNA from lung homogenates. The process of PCR was carried out referred to our previous study [29]. Briefly, the first-strand cDNA was generated from 4 μg of total RNA using oligo-dT to prime the reverse transcription reaction. The PCR mixture consisted of 10.4 μl of 2 × SYBR Green 1 Master Mix, 0.4 μl of both sense and antisense primers, 2.0 μl of sample cDNA solution, and distilled water to a final volume of 20 μl. Mouse β-actin was used as an internal control. The mRNA levels were normalized to the β-actin expression level and expressed using the comparative parameter threshold cycle (Ct). The PCR primers were bought from Shanghai Bioengineering Ltd (Shanghai, China). Table 1 shows all primers’ sequences that were used in our study.

**Table 1:** Sequence for primers in the present study

Gene name		Primer sequence
Mouse TNF-α	Forward	5′-TGATCCGCGACGTGGAA-3′
	Reverse	5′-ACCGCCTGGAGTTCTGGAA-3′
Mouse IL-1β	Forward	5′- ACTCCTTAGTCCTCGGCCA -3′
	Reverse	5′-CCATCAGAGGCAAGGAGGAA-3′
Mouse IFN-γ	Forward	5′- CTGGAGGAACTGGCAAAAGGATGG-3′
	Reverse	5′-GACGCTTATGTTGTTGCTGATGGC-3′
Mouse β-actin	Forward	5′-GGCTGTATTCCCCTCCATC-3′
	Reverse	5′-ATGCCATGTTCAATGGGGTA-3′

### Scratch wound healing assay

Human airway smooth muscle (HASM) cells were plated at 2 × 10^5^ cells/well into 6-well plates. The cells monolayer was scratched manually with a yellow plastic pipette tip, and washed with PBS to remove cell debris. Then, the cells were pretreated with or without DC260126 for 30 min, followed by incubation with oleic acid (OA) for another 24 h. The photographs of the scratch wound were recorded at 0 and 24 h to investigate and analyze the scratch wound assay using different samples. Digital photographs were obtained using an inverted microscope (Olympus, Japan), and the scratch area was measured using the Image-J software. The results were expressed as Migration aera (%) = (initial wound area at hour zero—the remaining wounded area) / initial wound area at hour zero.

### Cell cycle analysis

HASM cells were seeded in a 6-well tissue culture plate (2 × 10^5 ^cells/well), after treatment, the cells were collected and washed with PBS, and fixed in 1.5 ml 95% ethanol at 4 °C overnight, followed by incubation with RNase and staining with propidium iodide (Multi Sciences Biotech Co., Ltd) for 30 min. The DNA content was detected using a Cytomics FC500 Flow Cytometer (Beckman Coulter, USA). The percentage of cells in the G1 phase, the S phase, and the G2 phase was analyze.

### ELISA assay

Mice serum and BALFs were collected at 24 h after the last OVA challenge. The indicators (IL-4, IL-5, IL-13 in serum, and IL-1β, TNF-α, IFN-γ in BALFs) were separately detected by ELISA kits following the manufacturer's instruction. A Bio-Rad microplate reader was used to measure the absorbance of the tested sample at 450 nm.

### RhoA pull-down activation assay

GTP-bound RhoA protein measurement was carried out using RhoA activation assay kit (Cytoskeleton, Inc., USA). Briefly, lung tissues were fragmented and lysed in 1 × ice-cold assay/lysis buffer, and then centrifuged. Each sample's supernatant was fixed with 40 μl of either rhotekin RBD or PAK PBD agarose bead slurry, and the mixture was incubated at 4 °C for 1 h with slow agitation. Afterwards, the agarose beads were centrifuged, pelleted, and then resuspended after washed for 3 times. The precipitated GTP-RhoA expression was detected by western blot using anti-RhoA antibody.

### Western blot analysis

Briefly, the lung samples were fragmented and lysed in RIPA buffer (Beyotime, Biotechnology, Shanghai, China) containing 1% PMSF (Haoxin Biotechnology, Hangzhou, China). Sample protein (40 µg) were separated on 8–10% Tris/Glycine SDS-PAGE gel and subsequently transferred to PVDF membrane. Followed by 5% fat-free milk blocking for 1 h at room temperature, the blots were then incubated with primary Abs at 4℃ overnight. After rinsing in TBST, goat anti-rabbit 800 antibodies (1:5000) was used to probed the PVDF membranes. The immunoreactive bands were detected by a two-color infrared imaging system (Odyssey; LI-COR, Lincoln, NE, USA).

### Statistical analysis

The results are expressed as the mean ± S.E.M. One-way ANOVA followed by the Student–Newman–Keuls test was employed to determine multiple comparisons. All statistical calculations were performed using 18.0 SPSS software (Chicago, IL). *P* < 0.05 was considered statistically significance.

## Results

### GPR40 expression is elevated in the lung tissues of obese asthmatic mice

The experimental design of obese asthmatic model and GPR40 antagonist treatment procedure was presented in Fig. 1A. To investigate the GPR40 expression in obese asthmatic mice, lung tissues were harvested and assessed by immunochemical assay, as indicated in Fig. 1B, OVA challenge (*P* < 0.05) and HFD feeding (*P* < 0.05) could both significantly increased GPR40 expression, interestingly, obese asthmatic mice showed higher GPR40 expression (*P* < 0.01). As the previous study indicated, GRP40 is classified as the Gq-coupled receptor and is activated by medium- and long-chain FFAs [30]. Accordingly, the changes in FFAs expression in serum were evaluated, as shown in Fig. 1C, the level of FFAs in serum was significantly increased in HFD and HFD-OVA mice compared to control mice. The weight gain in the control and OVA group mice was not obvious, HFD and HFD-OVA group mice showed a significant weight gain during the 12 weeks of HFD feeding (Fig. 1D). To determine whether DC260126, a GPR40 antagonist, reduced body mass in an obese asthmatic model, mice were weighed with or without DC260126 intervention, as shown in Fig. 1E, higher body weight (∼32% higher) was observed in mice fed with a 45% fat diet (HFD and HFD-OVA group). Nevertheless, compared to the obese mice (39.07 ± 1.950 g) and obese asthmatic mice (38.35 ± 1.606 g), the body weight significantly decreased in mice that pretreated with DC260126 at 10 mg/kg (HFD + OVA + DC260126 10 mg/kg group) (35.78 ± 2.176 g) (*P* < 0. 05).

### GPR40 antagonist ameliorates AHR in obese asthmatic mice

**Fig. 2:**
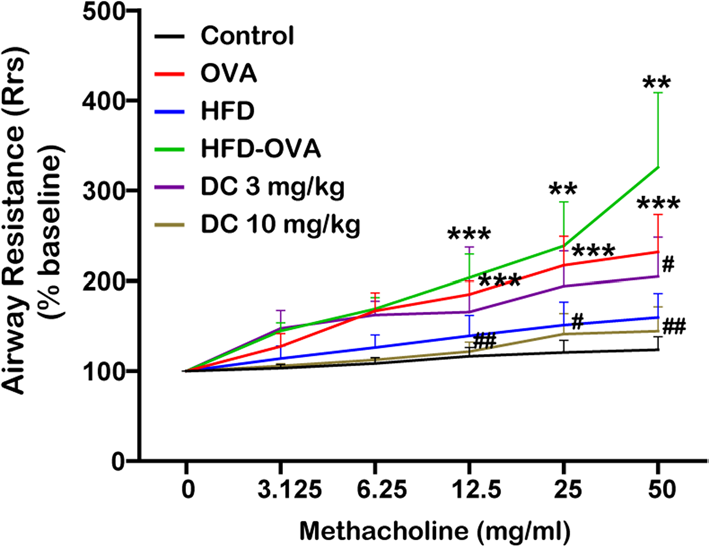
DC260126 alleviates methacholine-induced AHR in obese asthmatic mice. Within 24 h after last challenge, mouse ventilator and forced oscillation technique were employed to determine airway resistance. Airway resistance was measured by inhalation of methacholine at 0, 3.125, 6.25, 12.5, 25 and 50 mg/ml. The data were presented as the mean ± S.E.M (n = 6 per group). **P < 0.01 and ***P < 0.001 compared with the control, ^#^*P* < 0.05 and ^##^*P* < 0.01 compared with the HDF-OVA group

To study the effects of GPR40 antagonist on AHR, the changes of airway resistance (Rrs) after nebulizing with methacholine (0 to 50 mg/ml) were determined using the FlexiVent system (Fig. 2). Compared with the control mice, the animals in OVA and HFD model group presented a significant increase in Rrs starting from 25 to 50 mg/ml of methacholine. Moreover, we found there was a more pronounced increase in Rrs in HFD-OVA group mice in response to methacholine treatment (12.5 to 50 mg/ml), whereas, pretreatment with 3 or 10 mg/kg DC260126 to HFD-OVA mice could remarkably reduce these levels.

### GPR40 antagonist alleviates airway inflammation, collagen deposition and goblet cell hyperplasia in obese asthmatic mice

**Fig. 3:**
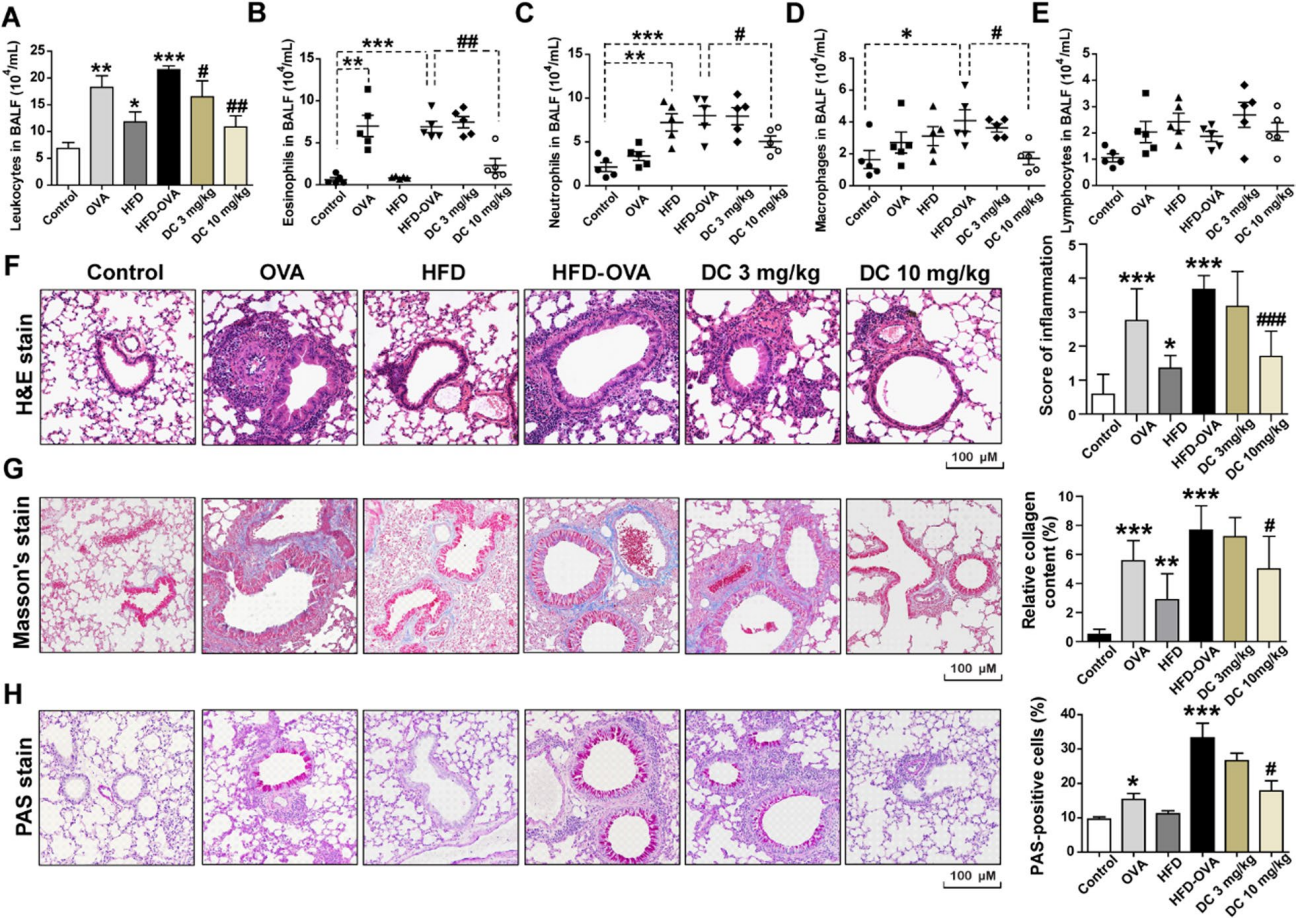
DC260126 ameliorates histopathological changes in obese asthmatic mice. The number of total inflammatory cells in BALFs were calculated (**A**), and a minimum of 200 cells were employed to classify eosinophils (**B**), macrophages (**C**), neutrophils (**D**) and lymphocytes (**E**) after the last OVA challenge (n = 6). The data were presented as the mean ± S.E.M. *P < 0.05, **P < 0.01 and ***P < 0.001 compared with the control, ^#^*P* < 0.05 and ^##^*P* < 0.01 compared with the HDF-OVA group. **F** Severity of inflammation cell infiltration in the peribronchiolar space was assessed by H&E staining, and semi-quantitative pathology scores among six groups were shown. **G** Lung sections were stained with Masson's trichrome stain for measurement of the subepithelial deposition of collagen and fibrosis, and staining analysis of collagen deposition was calculated (bar graph). **H** Lung sections were stained with PAS to assess goblet cell hyperplasia, and the percentage of PAS positive cells per bronchiole was calculated (histogram). The data were expressed as the mean ± S.E.M. (n = 6 per group). *P < 0.05, **P < 0.01 and ***P < 0.001 compared with the control, ^#^*P* < 0.05 and ^###^*P* < 0.001 compared with the HDF-OVA group

We next to evaluate the inhibitory effects of DC260126 on airway inflammation in obese asthmatic mice. Analysis of the BALFs showed a dramatic increase in total number of inflammatory cells (*P* < 0.001) in HFD-OVA group mice (Fig. 3A), which represented a significant increase in eosinophils (*P* < 0.001), neutrophils (*P* < 0.001) and macrophages (*P* < 0.05), but no elevation was detected in the number of lymphocytes. Pretreatment with DC260126 at 10 mg/kg, but not 3 mg/kg, significantly reduced the numbers of total leucocytes (*P* < 0.01), eosinophils (*P* < 0.01), neutrophils (*P* < 0.05) and macrophages (*P* < 0.05) (Fig. 3B–E). In addition, the inflammatory cells infiltration was assessed by H&E staining and graded based on histologic scoring system. Compared to the control mice, there was a significant higher inflammatory cells infiltration in the peribronchiolar space in OVA group, HFD group, and HFD-OVA group, whereas, pretreatment with DC260126 at 3 mg/kg (*P* < 0.05) or 10 mg/kg (*P* < 0.001) to HFD-OVA mice significantly alleviated the inflammatory cell infiltration (Fig. 3F). To determine the effects of DC260126 on collagen deposition, Masson's straining was employed. As presented in Fig. 3G, a significant accumulation of collagen deposition (blue staining) around the bronchi was observed in the HFD-OVA group (*P* < 0.001), DC260126 at 10 mg/kg could markedly ameliorate these pathological changes (*P* < 0.05). Furthermore, PAS staining was employed to assess goblet cell hyperplasia in the airway epithelium (Fig. 3H), compared to the control, the percentage of positive cell was significantly increased in the HFD-OVA group (*P* < 0.01), but this increase was greatly weakened by DC260126 pretreatment at 10 mg/kg (*P* < 0.05).

### GPR40 antagonist down-regulated cytokines in the lung of obese asthmatic mice

**Fig. 4:**
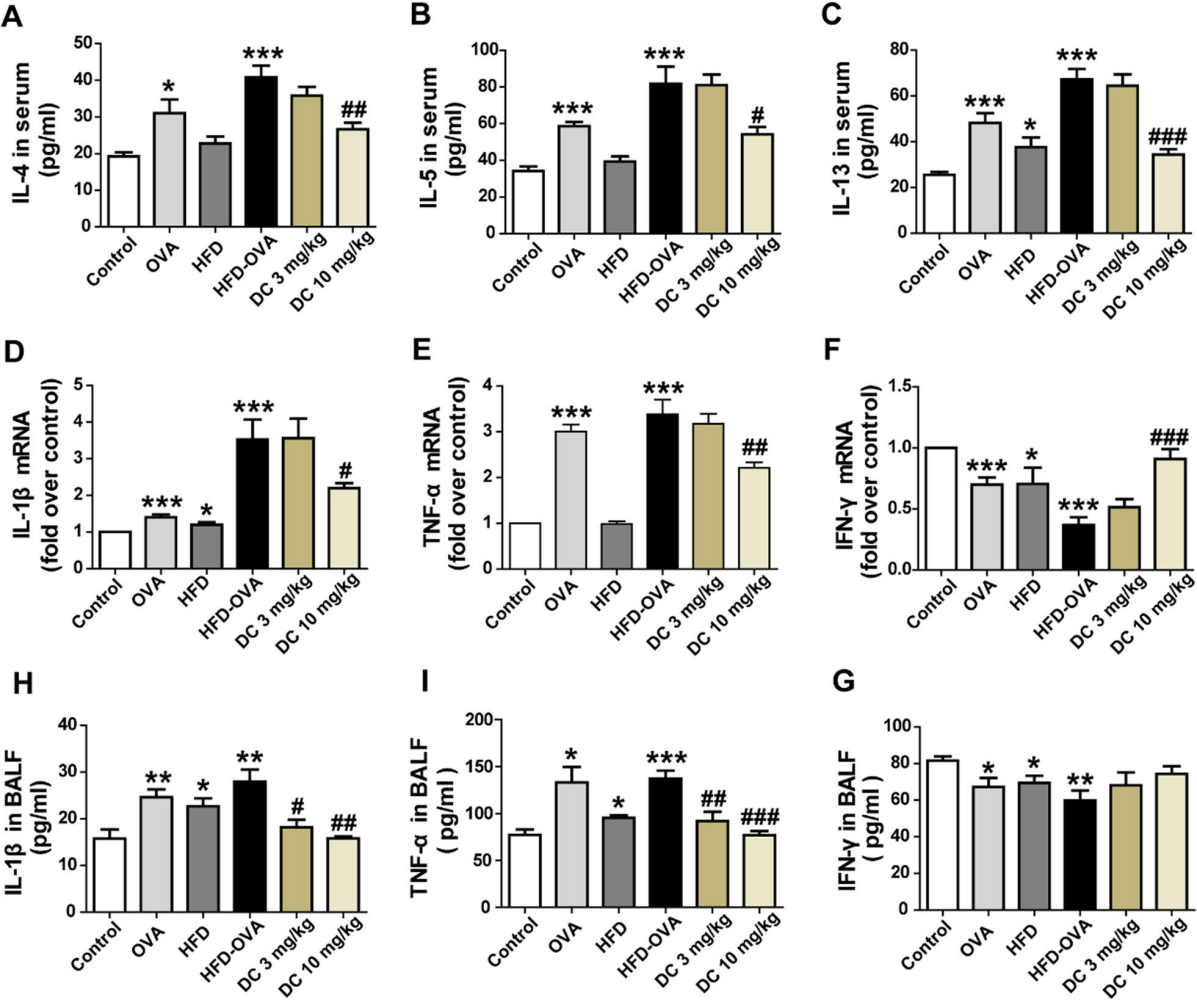
DC260126 down-regulated cytokines in the lung of obese asthmatic mice (**A**) The protein expression of IL-4 (**A**), IL-5 (**B**), and IL-13 (**C**) in serum was detected by ELISA assay (n = 6 per group). The gene expressions of IL-1β (**D**), TNF-α (**E**) and IFN-γ (**F**) in lung tissue was assessed by real-time PCR (n = 6 per group). The protein secretion of IL-1β (**H**), TNF-α (**I**) and IFN-γ (**G**) in BALFs was detected by ELISA assay (n = 6 per group). The data represent the mean ± S.E.M. from three independent experiments. *P < 0.05, **P < 0.01 and ***P < 0.001 compared with the control, ^#^*P* < 0.05, ^##^*P* < 0.01 and ^##^*P* < 0.001 compared with the HDF-OVA group

Furthermore, the changes in Th1, Th2 cytokines and pro-inflammatory cytokines were assessed. The levels of IL-4, IL-5, and IL-13 in serum were significantly increased in OVA and HFD-OVA mice compared to the control mice, however, these increases were significantly reduced in 10 mg/kg DC260126 treatment group (Fig. 4A–C). Interestingly, the gene expression of IL-1β (*P* < 0.001), TNF-α (*P* < 0.001) were up-regulated, and IFN-γ (*P* < 0.001) was down-regulated in HFD-OVA mice compared to the control, pretreatment with DC260126 could significantly reduce IL-1β, TNF-α and elevated IFN-γ expression in the lung tissue of mice (Fig. 4D–F). Using ELISA assay, we further confirmed that the mice in HFD-OVA group secreted a greater amount of IL-1β (*P* < 0.01), TNF-α (*P* < 0.001) and fewer amount of IFN-γ (*P* < 0.01) compared to the control group, the levels of IL-1β and TNF-α could be markedly reversed in 10 mg/kg DC260126 treatment group, but IFN-γ showed a similar trend that did not reach statistical significance (Fig. 4H–G).

### GPR40 antagonist reduces OA-induced cell proliferation and migration in HASM cells

**Fig. 5:**
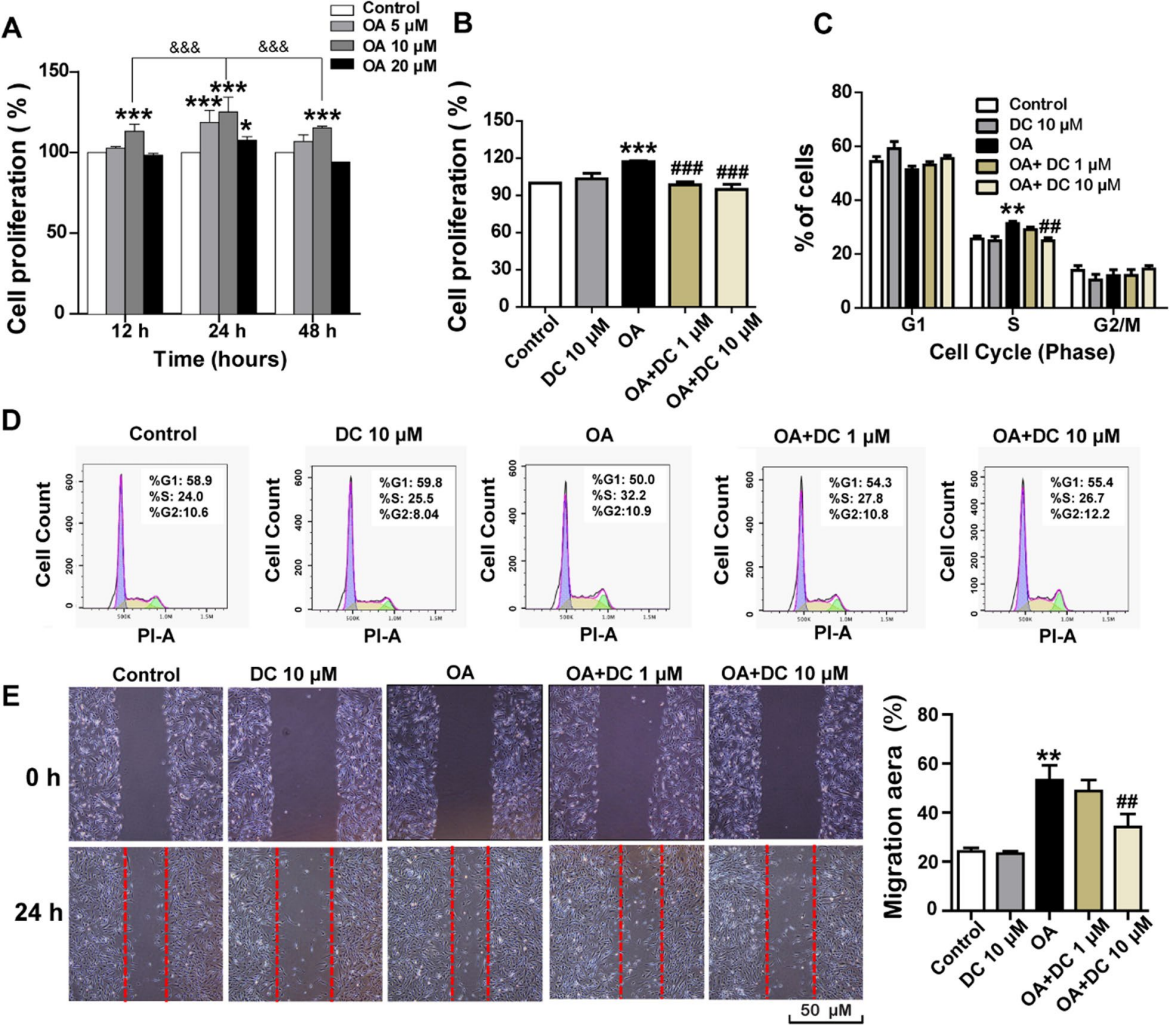
DC260126 inhibits OA-induced cell proliferation and migration in HASM cells*.* **A** The HASM cells were stimulated with oleic acid (OA) (0–20 µM) for 12 h, 24 h or 48 h, the cell proliferation was measured by CCK-8 kit. The data are expressed as the mean ± S.E.M. *P < 0.05 and ***P < 0.001 compared with the control. The percent of cell proliferation differences between the groups was done by two-way ANOVA. ^&&&^*P* < 0.001 compared with the 10 µM OA treatment at 24 h. **B** The cells were pretreated with 1 μM and 10 μM DC260126 for 0.5 h, then 24 h OA (10 μM)-induced cell proliferation was measured by CCK-8 kit (n = 8 per group). **C**, **D** The cells were pretreated with 1 μM and 10 μM DC260126 for 0.5 h, the effects of OA (10 µM) on cell cycle were assessed by flow cytometry. Representative DNA fluorescence histograms of propidium iodide (PI)-stained cells and statistical results were shown. **E** The cells were pretreated with 1 µM and 10 µM DC206126 for 0.5 h, the cell migration that accelerated by 10 μM OA was measured by scratch wound healing assay. The data are expressed as the mean ± S.E.M. (n = 4) from three independent experiments. **P < 0.01 and ***P < 0.001 compared with the control, ^##^*P* < 0.01 and ^###^*P* < 00.01compared with the OA-treated group

In the airways, GRP40 has been identified on airway smooth muscle cells and is reported to induce cell proliferation [31]. Herein, we evaluated the effects of oleic acid (OA), a representative long-chain FFAs, on HASM cells proliferation according to the previous research [19]. The HASM cell suspension that digested from the same culture flask was planted into three 96-well plates, then the cell proliferation assay was separately assessed at the time point of 12 h, 24 h,48 h. As presented in Fig. 5A, the 12 h, 24 h and 48 h exposure of HASM cells to OA (10 µM) resulted in a pronounced cell proliferation, whereas, the most obvious effects were observed at the 24 h time point. The results in Fig. 5B showed that the cell proliferation that induced by 10 µM OA at 24 h could be greatly suppressed by 0.5 h pretreatment of DC206126 at 1 µM (*P* < 0.001) and 10 µM (*P* < 0.001). Moreover, flow cytometry was employed to detect the effects of DC206126 on cell cycle distribution, we treated HASM cells with OA (10 μM) to induce cell cycle arrest at S phase (*P* < 0.01), but 10 μM DC206126 pretreatment caused a significant reduction of accumulated cells in S phase (Fig. 5C, D). By using a scratch wound model assay, we next examined whether blockage of GRP40 expression is involved in the alleviation of HASM cell migration, as shown in Fig. 5E, OA at 10 µM (*P* < 0.01) accelerated the cell migration after mechanical injury, pretreatment with DC206126 at 10 µM (*P* < 0.01) significantly inhibited the OA-induced cell migration.

### Targeting GPR40 expression with its antagonist alleviates obese asthma via RhoA/ROCK1 signaling pathway

**Fig. 6:**
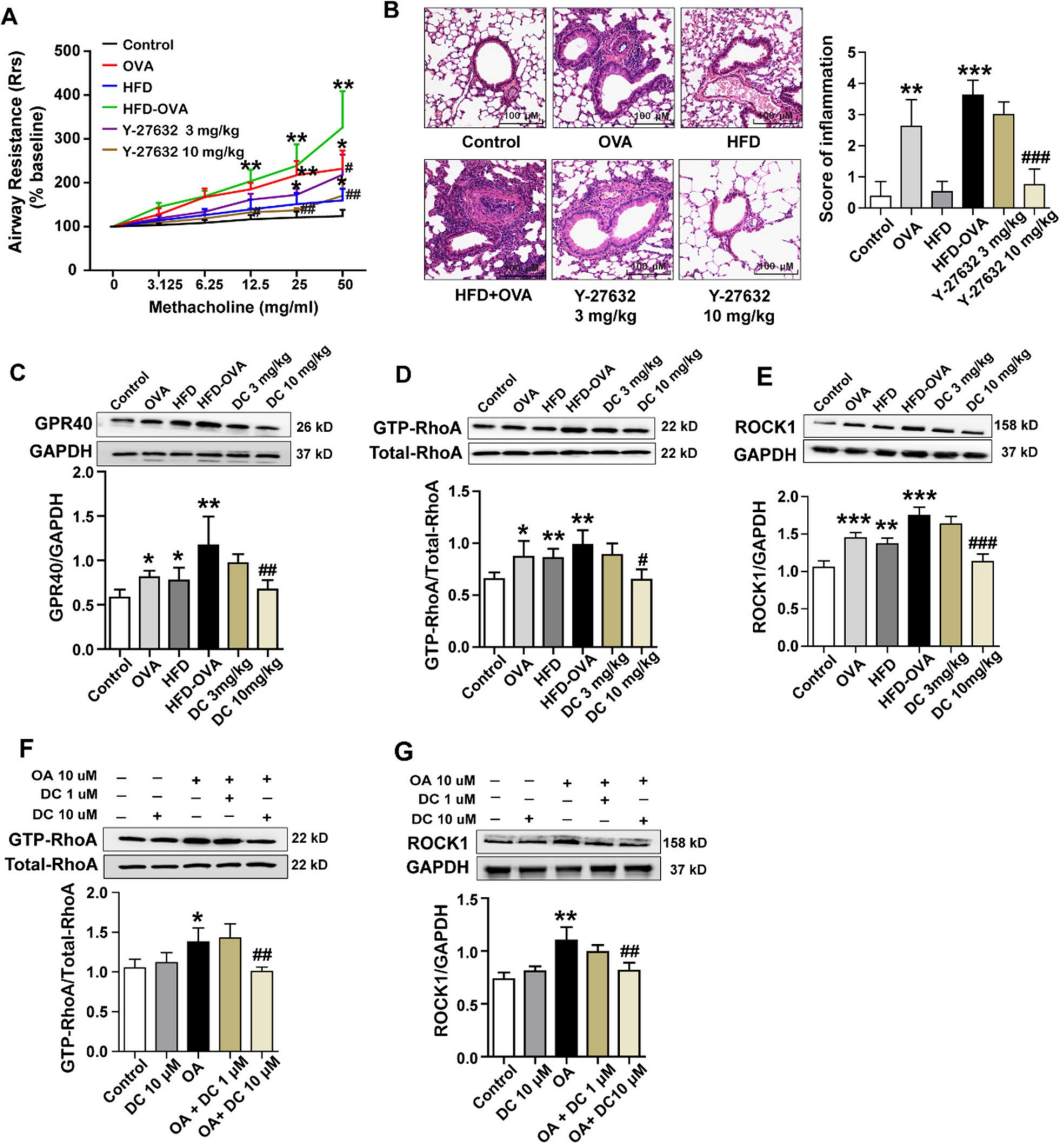
DC260126 alleviates AHR in obese asthmatic mice through RhoA/ROCK1 signal pathway. **A** Within 24 h after last challenge, airway resistance was measured by inhalation of methacholine (0 to 50 mg/ml) to determine airway resistance (n = 6 per group). **B** Severity of inflammation cell infiltration in the airways was assessed by H&E staining, and semi-quantitative pathology scores were shown. Mouse lung tissues from different treatment groups were harvested, the protein expression of GPR40 (**C**), the activity of RhoA (GTP-RhoA) (**D**) and protein expression ROCK1 (**E**) were evaluated by western blot (n = 4 per group). The data were presented as the mean ± S.E.M. *P < 0.05, **P < 0.01 and ***P < 0.001 compared with the control, ^#^*P* < 0.05, ^##^*P* < 0.01 and ^###^*P* < 0.001 compared with the HDF-OVA group. The cell lysates of HASM cells from different treatment groups were harvested, the activity of RhoA (GTP-RhoA) (**F**) and protein expression of ROCK1 (**G**) were assessed by western blot (n = 4 per group). The data were presented as the mean ± S.E.M. *P < 0.05, **P < 0.01 compared with the control, ^##^*P* < 0.01 compared with the OA-treated group

We then explored whether the RhoA/ROCK1 signaling pathway was involved in GPR40-regulated asthma in obese mice. We first evaluated the inhibitory effects of a specific inhibitor of Rho-associated kinases, Y-27632, on AHR, and found 10 mg/kg Y-27632 pretreatment resulted in a pronounced reduction in the airway resistance (Rrs) that elevated in HFD-OVA group (Fig. 6A). Furthermore, H&E staining revealed that inflammatory cells infiltration in the peribronchiolar space in HFD-OVA group was significantly alleviated by the pretreatment of Y-27632 at 10 mg/kg (*P* < 0.01) (Fig. 6B). Additionally, we found that GRP40 was greatly expressed in the lung of HFD-OVA group, but could be drastically down-regulated by 10 mg/kg DC260126 pretreatment (*P* < 0.01) (Fig. 6C). As shown in Fig. 6D and E, DC260126 at10 mg/kg remarkedly suppressed both the GTP-RhoA (*P* < 0.001) and ROCK1 expression (*P* < 0.001) in mouse lungs of HFD-OVA group in vivo. Furthermore, we found the increased GTP-RhoA (*P* < 0.01) (Fig. 6F) and ROCK1 (*P* < 0.01) (Fig. 6G) levels that triggered by 10 μM OA could be down-regulated by DC260126 pretreatment (*P* < 0.01) in vitro*.* These data underscored that GPR40 regulated the RhoA/ROCK1 signaling pathway in obese asthmatic mice.

## Discussion

In this article, we demonstrated that targeting of GPR40 with its small molecule antagonist, DC260126, effectively alleviated AHR, ameliorated airway inflammation and pulmonary pathological changes in obese asthmatic mice. Moreover, DC260126 reduced OA-induced HASM cells proliferation and migration in vitro*.* Mechanistically, we found that DC260126 alleviated AHR in mice model and inhibited airway smooth muscle cell proliferation associated with the RhoA/ROCK1 signaling pathway. As far as we know, it is the first report to determine the effects of GPR40 antagonist on obese asthma, and thus suggest GPR40 may be a potent molecular target for obese asthma treatment.

FFAs, especially the long-chain FFAs, have been indicated to participate in the development of many metabolic diseases including obesity, diabetes, and atherosclerosis [32]. GPR40, which also called FFAR1, worked as the sensor of medium- to long-chain FFAs [33]. GPR40 was indicated to express on airway smooth muscle and contribute to airway smooth muscle contraction which could worsen asthma symptoms [34]. In the present study, the discovery of elevated FFAs level in the serum of HFD-fed mice, and high GRP40 expression on airways urged us to consider whether the receptor could regulate AHR in obese asthma. In airway smooth muscle, the changes in intracellular Ca2 + concentration ([Ca2 +]i) and Ca2 + oscillations are required for sustained airway contraction [35]. GPR40 is generally considered to be a Gq coupled receptor, the downregulation of which by gene knockdown caused a significant inhibition of the long-chain FFAs (oleic acid and linoleic acid)-induced transient [Ca2 +]i increases and airway smooth muscle contraction [19]. Airway smooth muscle was recognized as the main cell type responsible for AHR [36], and the changes in airway smooth muscle contractile properties play an important role in the development of AHR in asthma [37]. By employing DC260126, the small molecule antagonist of GPR40, we proved that the body weight in HFD-OVA was greatly downregulated, and DC260126 could reduce the airway resistance in obese asthma. Moreover, increased airway smooth muscle (ASM) mass has been deemed as a key contributor to airway narrowing and AHR in asthma [38]. In cultured HASM cells, we found that DC260126 could remarkedly alleviate oleic acid (OA)-induced cell proliferation and migration. Our findings imply that GPR40 played a pivotal role in regulating AHR in obese asthma.

Some asthma patients are reported to develop AHR in association with airway remodeling [39]. Airway remodeling, which comprises several features including: increased sensitivity, hypertrophy and hyperplasia of smooth muscle cells, as well as increased subepithelial fibrosis characterized by increased collagen could lead to persistently altered airway wall structures and lung mechanics [40]. In the present study, significant histopathological changes were detected in obese asthma, which supported by the finding of some previous study that obesity-associated factors can cause inflammation and remodeling of the airway. Moreover, an influx of eosinophils, neutrophils and macrophages were detected in both BALFs and lung parenchyma of obese asthma animals. Th2-dominant eosinophils are the most characteristic inflammatory cells in the airway mucosa in the OVA model, which are closely related to the asthmatic symptoms, and AHR [41]. However, in the obese asthmatic mice in the present study, the elevated levels of Th2 cytokines (IL-4, IL-5, and IL-13) and pro-inflammatory cytokines (IL-1β, TNF-α) with modest inflammation were observed, on the contrary, the Th1 cytokine (IFN-γ) expression was significantly decreased. Moreover, we found that the DC260126-treated mice presented lower levels of IL‐4, IL‐5, IL-13 and reduced accumulation of eosinophils in the lungs. By signaling predominantly through Gαq/11, GPR40 increases intracellular Ca2 + concentration and participates in insulin secretion [42]. Mice deficient in Gq were previously shown to have impaired eosinophil recruitment to lungs in asthmatic mice [16]. Based these effects, DC260126 alleviated eosinophilic inflammation probably through blunted the recruitment of eosinophil to the lung and airway in obese asthma, however, which would be further investigated in our following study. IFN‐γ could inhibit the recruitment of eosinophils by inhibiting CD4 + T cell infiltration, reducing IL‐4 and IL‐5 production [43]. It was demonstrated that IFN‐γ was greatly decreased in asthma group, and the concentration was the lowest in obese asthma mice [44], which is consistent with our results. Additionally, we detected that DC260126 markedly reversed the changes of IFN-γ gene expression, but not the protein expression, which probablely be improved if a relatively long duration of treatment was employed. Although, the role of inflammation in obesity is controversial in the lungs, sputum neutrophils in obese asthmatics have been recently reported to increased greatly [45]. Increased circulating adipokines, as well as elevated levels of IL-1β and TNF-α are associated with asthma in obese individuals, but not with allergic asthma [9, 46]. Moreover, IL-1β and TNF-α have been indicated to play a prominent role in the development of AHR and neutrophilic airway inflammation [47, 48]. TNF-α blockade or depletion of alveolar macrophages in the obese mice could decrease AHR [49]. Ma et al. found that blockade of IL-1β with an IL-1 receptor antagonist could also abolish obesity-induced AHR [50]. Importantly, GPR40 was found up-regulated on neutrophils under inflammatory settings, and plays a role in regulating the inflammatory response [51]. In line with the previous studies, the levels of IL-1β and TNF-α in our study were greatly up-regulated in HFD-OVA group mice, pretreated with DC260126 could down-regulated the IL-1β and TNF-α expression. Considering the great inhibitory effects of DC260126 on Th1, Th2 cytokines and pro-inflammatory cytokines expression in obese asthma, it is of great significance to validate the potential use of GPR40 as anti-obesity-associated asthma drug target.

RhoA is an important molecule for mediating cell signal transduction, which could combine with its downstream target molecule, ROCK1, to directly modulate contractile proteins to participate in actin cytoskeleton reconstruction [52]. RhoA/ROCK1 pathway has been reported to link closely with AHR in asthma [53]. Activation of RhoA that markedly augmented Ca2 + sensitization was deemed to play a vital role in the contraction of airway smooth muscle [54]. On the other hand, the Rho-kinase inhibitor, Y-27632, was indicated to attenuate airway hyperresponsiveness, inflammation, and the extracellular matrix remodeling in an asthma model [55].. Moreover, RhoA/ROCK1 signaling pathway has been demonstrated to be implicated in diverse metabolic functions throughout the body, which could be greatly increased in adipose tissue of diet-induced or genetically obese mice [24]. Importantly, RhoA has been reported to locate downstream of numerous GPCRs and serve as the proximal effector to regulate a variety of basic cell function [23]. To determine whether GPR40 regulates the RhoA/ROCK in obese asthma in our study, we showed that there was a significant increased expression of GTP-RhoA and ROCK1 in the lung tissues of obese asthmatic mice and OA-treated HASM cells, whereas, targeting GPR40 with DC260126 markedly suppressed GTP-RhoA and ROCK1 expression both in vivo and in vitro. The above findings remind us that RhoA/ROCK signal pathway is an important mechanism that GPR40 regulated obese asthma.

## Conclusion

In summary, we demonstrated that DC260126, a GPR40 antagonist, shows anti-asthmatic effects on airway inflammatory infiltration, AHR and pathological changes in obese mice. Furthermore, DC260126 greatly down-regulated the activation of RhoA and ROCK1, thus suggesting that GPR40 was associated with the regulation of RhoA/ROCK1 signaling pathway in obese asthmatic mice. Based on our findings, we suggest that GPR40 antagonist may be a potential candidate for the pharmacological therapy of obese asthma.

## Supplementary Information

**Additional file 1: Fig. S1.** Effects of GPR40 inhibition on airway inflammation in asthmatic mice. (A) The number of total inflammatory cells in BALFs were calculated, and a minimum of 200 cells were employed to classify eosinophils (A), Neutrophils (B), macrophages (C) macrophages (D) and lymphocytes (E) after the last OVA challenge. BALFs were harvested to measure IL-4 (F), IL-13 (G) and IL-8 (H) release by ELISA. The data are expressed as the mean ± S.E.M (n=6). *P<0.05, **P<0.01 and ***P<0.001 compared with the control group, ^#^*P*<0.05, ^##^*P*<0.01 and ^###^*P*<0.001 compared with the OVA group. **Fig. S2.** Mouse lung tissues were collected for the extraction of protein. The expression of GPR40 was measured by western blot. The data are expressed as the mean ± S.E.M (n=4). **P<0.01 compared with the control group, ^##^*P*<0.01 compared with the OVA model group.

## Data Availability

The analyzed datasets generated during the study are available from the corresponding author on reasonable request.

## Abbreviations

AHR: Airway hyperresponsiveness
GPR40: G-protein coupled receptor 40
HFD: High-fat diet
RhoA: Ras homolog family member A
ROCK1: Rho-associated coiled-coil-forming protein kinase 1
FFAs: Free fatty acids
ASM: Airway smooth muscle
HASM: Human airway smooth muscle
ASM: Airway smooth muscle
Rrs: Airway resistance
OVA: Ovalbumin
BALFs: Bronchoalveolar lavage fluids
Mch: Methacholine
GPCRs: G Protein-Coupled Receptors
DMSO: Dimethyl sulfoxide
CCK-8: Cell Counting Kit-8
IL-4: Interleukin-4
IL-5: Interleukin-5
IL-13: Interleukin-13
IL-1β: Interleukin-1β
DC: DC260126
OA: Oleic acid
