# Supplementary material for: Targeting of G-protein coupled receptor 40 alleviates airway hyperresponsiveness through RhoA/ROCK1 signaling pathway in obese asthmatic mice
Source: Respir Res. 2023 Feb 17;24:56. doi: 10.1186/s12931-023-02361-1 (PMC9938616; doi:10.1186/s12931-023-02361-1)
Supplement: Supplementary file 1 — Additional file 1: Fig. S1. Effects of GPR40 inhibition on airway inflammation in asthmatic mice. (A) The number of total inflammatory cells in BALFs were calculated, and a minimum of 200 cells were employed to classify eosinophils (A), Neutrophils (B), macrophages (C) macrophages (D) and lymphocytes (E) after the last OVA challenge. BALFs were harvested to measure IL-4 (F), IL-13 (G) and IL-8 (H) release by ELISA. The data are expressed as the mean ± S.E.M (n=6). *P<0.05, **P<0.01 and ***P<0.001 compared with the control group, #P<0.05, ##P<0.01 and ###P<0.001 compared with the OVA group. Fig. S2. Mouse lung tissues were collected for the extraction of protein. The expression of GPR40 was measured by western blot. The data are expressed as the mean ± S.E.M (n=4). **P<0.01 compared with the control group, ##P<0.01 compared with the OVA model group. [file 12931_2023_2361_MOESM1_ESM.docx]

1. ***DC260126 at high dose causes neutrophilic airway inflammation in asthma***

We first evaluate the inhibitory effects of DC260126 on OVA-induced airway inflammation. As shown in the Figure S1. A-E, compared with the asthma group, DC260126 at 10 mg/kg, but not 30 mg/kg, presented a marked reduction in the number of the total inflammatory cell, lymphocytes and macrophages in the BALFs. Unexpectedly, DC260126 at 30 mg/kg is able to greatly reduce the eosinophils number, but obviously up-regulated the number of neutrophils in asthmatic mice, which implied that DC260126 at high dose probably aggravated the neutrophilic airway inflammation.

Furthermore, we attempted to explore whether inhibition of GPR40 expression reduced Th2 cytokine and IL-8 levels in asthmatic mice. Our data suggested that DC260126 at 10 mg/kg noticeably down-regulated the protein levels of IL-4, IL-13 and IL-8 when compared to the model group mice (Figure S1. F-H), however, DC260126 at 30 mg/kg failed to exert a stronger lowering effect as expected.

**
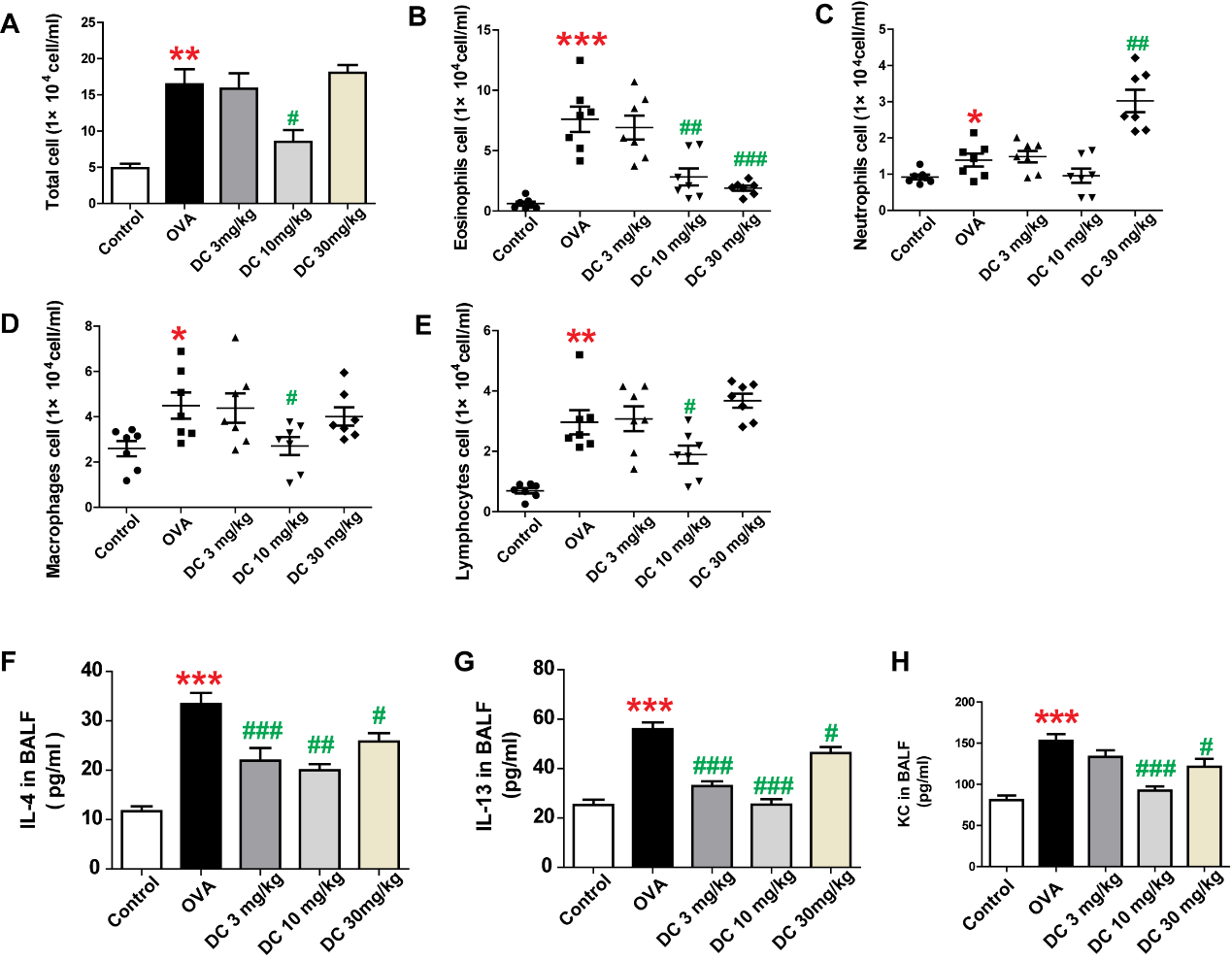
**

**Fig. S1 Effects of GPR40 inhibition on airway inflammation in asthmatic mice.** (A) The number of total inflammatory cells in BALFs were calculated, and a minimum of 200 cells were employed to classify eosinophils (A), Neutrophils (B), macrophages (C) macrophages (D) and lymphocytes (E) after the last OVA challenge. BALFs were harvested to measure IL-4 (F), IL-13 (G) and IL-8 (H) release by ELISA. The data are expressed as the mean ± S.E.M (n=6­). **P*<0.05, ***P*<0.01 and ****P*<0.001 compared with the control group, ^#^*P*<0.05, ^##^*P*<0.01 and ^###^*P*<0.001 compared with the OVA group.

1. ***DC260126 at 10 mg/kg greatly inhibits GPR40 expression in asthma***

To confirm the doses of DC260126 at 3 and 10 mg/kg are sufficient to provide an antagonist effect on GPR40, we have also evaluated the protein expression of GPR40 in an asthma model, and found GRP40 was greatly expressed in the lung of OVA group, but could be drastically down-regulated by 10 mg/kg DC260126 pretreatment (Figure S2).


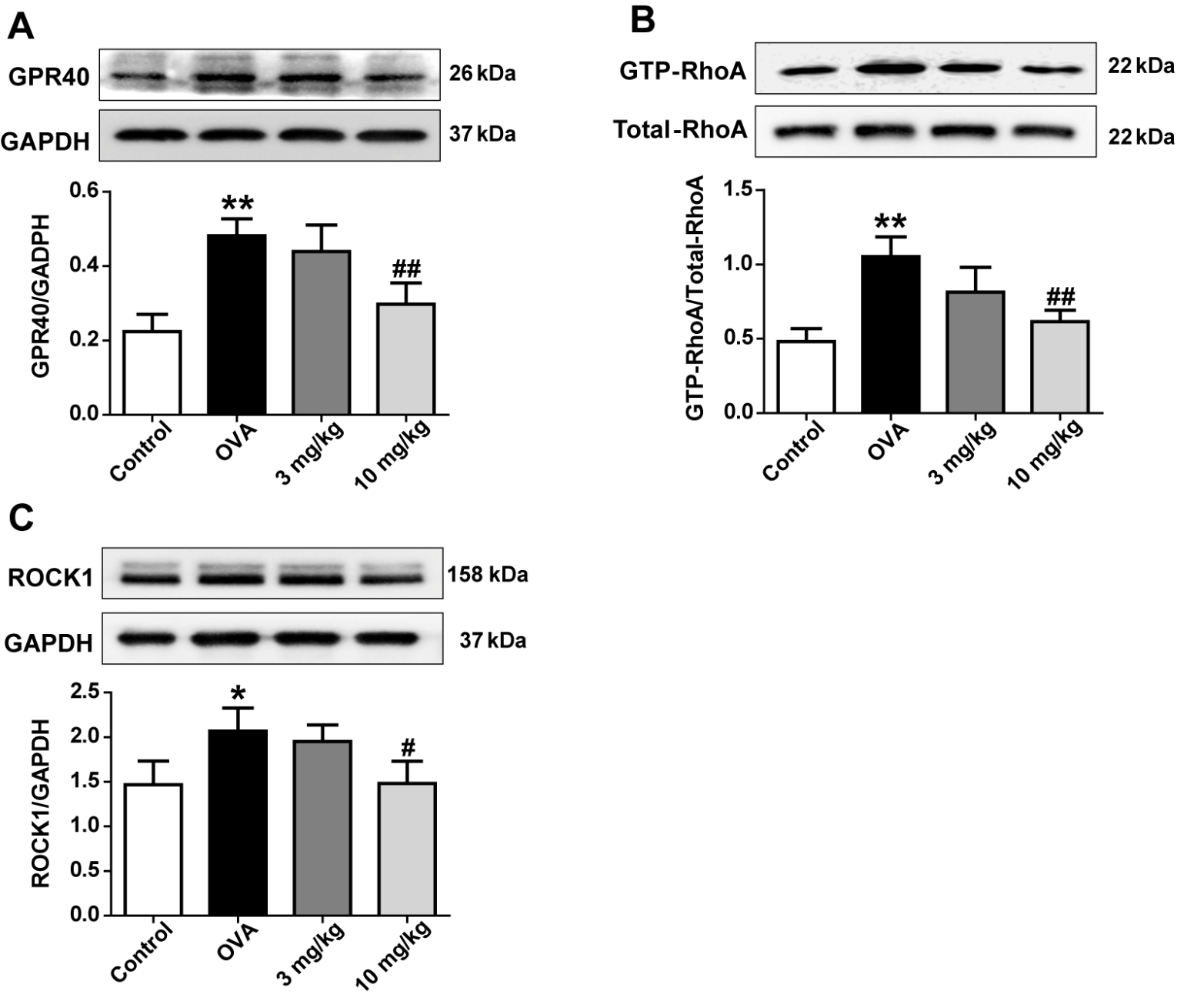


**Fig. S2** Mouse lung tissues were collected for the extraction of protein. The expression of GPR40 was measured by western blot. The data are expressed as the mean ± S.E.M (n=4­). ***P*<0.01 compared with the control group, ^##^*P*<0.01 compared with the OVA model group.
